# Contrasting trait syndromes in angiosperms and conifers are associated with different responses of tree growth to temperature on a large scale

**DOI:** 10.3389/fpls.2013.00409

**Published:** 2013-10-17

**Authors:** Jofre Carnicer, Adrià Barbeta, Dominik Sperlich, Marta Coll, Josep Peñuelas

**Affiliations:** ^1^Community and Conservation Ecology Group, Centre for Ecological and Evolutionary Studies, University of GroningenGroningen, Netherlands; ^2^CREAFBarcelona, Spain; ^3^Global Ecology Unit, Consejo Superior de Investigaciones Científicas, CREAF-CEAB-CSIC-UABBarcelona, Spain; ^4^Department of Ecology, University of BarcelonaBarcelona, Spain

**Keywords:** conifers, angiosperms, functional traits, mediterranean ecosystems, drought, temperature, carbon metabolism, growth

## Abstract

Recent large-scale studies of tree growth in the Iberian Peninsula reported contrasting positive and negative effects of temperature in Mediterranean angiosperms and conifers. Here we review the different hypotheses that may explain these trends and propose that the observed contrasting responses of tree growth to temperature in this region could be associated with a continuum of trait differences between angiosperms and conifers. Angiosperm and conifer trees differ in the effects of phenology in their productivity, in their growth allometry, and in their sensitivity to competition. Moreover, angiosperms and conifers significantly differ in hydraulic safety margins, sensitivity of stomatal conductance to vapor-pressure deficit (VPD), xylem recovery capacity or the rate of carbon transfer. These differences could be explained by key features of the xylem such as non-structural carbohydrate content (NSC), wood parenchymal fraction or wood capacitance. We suggest that the reviewed trait differences define two contrasting ecophysiological strategies that may determine qualitatively different growth responses to increased temperature and drought. Improved reciprocal common garden experiments along altitudinal or latitudinal gradients would be key to quantify the relative importance of the different hypotheses reviewed. Finally, we show that warming impacts in this area occur in an ecological context characterized by the advance of forest succession and increased dominance of angiosperm trees over extensive areas. In this context, we examined the empirical relationships between the responses of tree growth to temperature and hydraulic safety margins in angiosperm and coniferous trees. Our findings suggest a future scenario in Mediterranean forests characterized by contrasting demographic responses in conifer and angiosperm trees to both temperature and forest succession, with increased dominance of angiosperm trees, and particularly negative impacts in pines.

## Introduction

The assimilation and allocation of carbon are fundamental processes allowing tree growth, development, survival, reproduction and defense (McDowell, 2011; Galiano et al., 2012; Sala et al., 2012). In addition, non-structural carbohydrates (NSCs) play a variety of functions in tree physiology, providing a temporal buffer to reconcile differences in carbon supply and demand, maintaining hydraulic transport and facilitating osmotic regulation, allowing leaf emergence and bud burst and actively participating in the prevention of frost and drought embolism and repair (Sala et al., 2012). The demographic performance of trees, however, is generally co-limited by other factors that frequently interact in complex ways with the processes of carbon uptake and allocation, such as direct climatic effects on photosynthesis, growth and nutrient uptake (Körner, 1998, 2003; Rennenberg et al., 2006), species-specific traits (Wright et al., 2004; Chave et al., 2009; Carnicer et al., 2012) or the impacts of secondary consumers and diseases (Bale et al., 2002).

Recent ecophysiological studies highlight the coupled dynamic links between NSC content in woody tissues and several climate-dependent tree responses such as embolism prevention and repair, growth, bud burst and leaf emergence (Johnson et al., 2012; Sala et al., 2012; Meinzer and McCulloh, 2013). These studies suggest the existence of contrasting trait-based ecophysiological strategies in major plant groups (Choat et al., 2012; Johnson et al., 2012; Meinzer et al., 2013) such as angiosperm and coniferous trees. Arguably, a next necessary step is to analyze how these contrasting ecophysiological strategies may be influencing the distribution and abundance of tree species and their responses to global warming.

Recent large-scale studies have reported contrasting responses of growth to temperature in angiosperm and coniferous trees in Mediterranean forests of the Iberian Peninsula (Gómez-Aparicio et al., 2011; Coll et al., 2013). For example, Gómez-Aparicio et al. (2011) reported a positive effect of rising temperatures on growth of angiosperm trees, but neutral or negative effects on coniferous trees. These contrasting trends between the two phylogenetic groups were later also observed and confirmed by Coll et al., (2013). Critically, whereas a reduction in precipitation was predicted to decrease tree growth in both groups, increases in temperature could produce a performance disadvantage in conifers compared to angiosperm broadleaved trees (Gómez-Aparicio et al., 2011; Coll et al., 2013). Consistent with these empirical findings that associate the negative effects of temperatures and growth in *Pinus* species, palaeoecological studies suggest a persistent link between Pinaceae distributions and low temperatures during the last 100 million years (Millar, 1993; Brodribb et al., 2012). Cold periods in the Paleocene and Eocene are associated with an increased abundance of fossils of the genus *Pinus*, and the reverse occurs during warm periods (Millar, 1993; Brodribb et al., 2012). Similarly, warm periods during the Miocene and Pliocene are apparently associated with northward contractions of the ranges of Pinaceae species (Millar, 1993; Brodribb et al., 2012). Notably, the ecophysiological basis of these contrasting growth and distributional responses to temperature remain poorly discussed and resolved.

Here we review the hypotheses that may contribute to explain the observed contrasting responses of growth to temperature observed in Mediterranean conifers and angiosperms. We review the differences between Mediterranean conifer and angiosperm trees in growth-related traits, including phenology, crown allometry, sensitivity to competition, and drought and winter freezing responses. Furthermore, we hypothesize that angiosperm and coniferous ecophysiological strategies differentially integrate diverse traits such as stomatal sensitivity to vapor-pressure deficit (VPD), hydraulic safety margins and capacity for embolism repair, which in turn are linked to features of the xylem such as NSC content, carbon transfer rates, wood parenchymal fraction and wood capacitance. In sum, our main aims in this study are: (i) to list the different hypotheses that may explain contrasting growth responses to temperature in Mediterranean conifer and angiosperm trees and review the differences in eco-physiological traits associated with temperature- and drought-induced responses in these two groups, (ii) to briefly review the multiple effects of temperature on basic tree ecophysiological functions (e.g., photosynthesis, growth, respiration and nutrient uptake and transport), (iii) to analyze the specific case study of forests in the Iberian Peninsula, which present diverging tree growth responses to temperature in Angiosperms and Conifers, and (iv) to briefly discuss the implications of our findings. Below we dedicate a section to each of these objectives.

## A review of the diverse hypotheses that may explain contrasting growth responses to temperature in mediterranean and angiosperm trees

Table 1 lists the different hypotheses that may explain contrasting growth trends to temperature in Mediterranean conifer and angiosperm trees. The first hypothesis (Table 1.1) states that positive growth responses to increased temperature in angiosperms could be mediated by a less strict stomatal control, allowing them to assimilate carbon for longer during warmer and drier periods. While this could imply that angiosperm could be more vulnerable to xylem cavitation and hydraulic failure, they have a greater capacity for embolism repair. On the other hand, most conifers function with a wider hydraulic safety margin to avoid cavitation but with the cost of lower carbon gain. Beside this specific hypothesis, several other factors could also contribute to explain the differences in growth responses between conifer and angiosperm trees. For example, these two groups differ in the effects of phenology in their productivity, in the sensitivity of growth to competition, and in growth allometry (Table 1.2–1.4). In addition, local adaptation processes and phenotypic plasticity also largely influence tree growth responses to temperature and drought (Table 1.5–1.9). Finally, the available empirical evidence suggests that the diverse factors significantly interact in determining growth responses (Table 1.7). For example, several studies report strong interactions between tree size, drought, and stand density effects in determining large-scale growth patterns in the Mediterranean basin. Below we briefly review the hypotheses listed in Table 1 and discuss the experimental tests required to assess their relative importance.

**Table 1:** **Main hypotheses that may contribute to explain contrasting growth responses to temperature in Iberian Angiosperm and Conifer trees on a large scale**.

**Hypotheses**	**Angiosperms**	**Conifers**	**References**
1.1 Eco-physiological and hydraulic traits	Narrower hydraulic safety margins and higher capacity to reverse embolisms	Wide hydraulic safety margins, early drought-induced stomatal closure and lower carbon gain, low stomatal conductance sensitivity to VPD	Martínez-Ferri et al., 2000; Brodersen et al., 2010; Choat et al., 2012; Epron et al., 2012; Johnson et al., 2012; Michelot et al., 2012; Sala et al., 2012; Brodersen and McElrone, 2013; Coll et al., 2013; Meinzer et al., 2013; Ogasa et al., 2013
1.2 Phenology	Tree productivity more sensitive to growing season length	Positively affected but less sensitive to growing season length	Churkina et al., 2005; Piao et al., 2007; Welp et al., 2007; Delpierre et al., 2009; Richardson et al., 2010; Gómez-Aparicio et al., 2011; Coll et al., 2013
1.3 Intra- and inter-specific competition and forest succession	Growth less sensitive to intra and inter-specific stand competition	Growth severely reduced by intra- and inter-specific competence in small, non-dominant trees	Sánchez-Gómez et al., 2008; Gómez-Aparicio et al., 2011; Carnicer et al., 2013a; Coll et al., 2013; Vayreda et al., 2013
1.4 Size, age and allometry	Different growth allometry and less apical dominance	Peak of crown growth reached at lower sizes	Gómez-Aparicio et al., 2011; Poorter et al., 2012
1.5 Drought and temperature	Angiosperm trees are able to maintain substantial transpiration levels during summer drought events	Drought and heat waves often results in early stomatal closure in Mediterranean conifers	Martínez-Ferri et al., 2000; de Luis et al., 2007, 2011; Zweifel et al., 2007; Eilmann et al., 2009; Camarero et al., 2010; Klein et al., 2011; n, Coll et al., 2013; Poyatos et al., 2013
1.6 Winter freezing	Angiosperm trees are more vulnerable to freeze-thaw embolism	Less sensitive to freeze-thaw embolism	Sperry and Sullivan, 1992; Gómez-Aparicio et al., 2011; Brodribb et al., 2012
1.7 Interactions between multiple factors	Yes	Yes	Linares et al., 2010; Gómez-Aparicio et al., 2011; Vayreda et al., 2012; Coll et al., 2013; Ruiz-Benito et al., 2013
1.8 Local adaptation, individual and provenance variation	Yes	Yes	Rehfeldt, 1978, 1982; Santos et al., 2010; Ramírez-Valiente et al., 2010, 2011; Chmura et al., 2011; Robson et al., 2012; Alberto et al., 2013
1.9 Phenotypic plasticity	Yes	Yes	Camarero et al., 2010; Nicotra et al., 2010; de Luis et al., 2011

### Eco-physiological and hydraulic traits. different ecophysiological and carbon-allocation strategies in angiosperms and conifers (Hypothesis 1.1)

Table 2 summarizes the trait differences between angiosperm and coniferous trees. Key traits that differ between these two groups include stomatal sensitivity to VPD, xylem anatomy, foliar traits, hydraulic safety margins, capacity for embolism repair, NSC content, carbon transfer rates, wood parenchymal fraction, and wood capacitance. The available published evidence shows that these diverse traits are functionally related and define two contrasting ecophysiological strategies in conifers and angiosperms. Compared to angiosperms, conifers have a lower stomatal-conductance sensitivity to increased VPD (*sensu* Johnson et al., 2012). In turn, this key difference in stomatal response appears to be tightly related to the different hydraulic safety margins in both groups (Tyree and Sperry, 1988; Nardini et al., 2001; Table 2). The wider hydraulic safety margins in conifers thus imply early responses of stomatal closure, which reduce hydraulic conductivity before substantial cavitation occurs. On the other hand, angiosperms can maintain relatively high stomatal conductances even when the xylem pressure caused by high VPD is sufficient to induce extensive cavitation (Meinzer et al., 2009, 2013; Johnson et al., 2012).

**Table 2:** **Summary of differences in key functional traits between conifers and angiosperms**.

**Trait**	**Angiosperms**	**Conifers**	**References**
Wood anatomy	Vessels	Tracheids	Brodribb et al., 2012
	Ring-porous and diffuse-porous		
	Homogeneous pit membrane	Torus-margo pit membrane	
	Cylindrical phloem sieve elements Companion cells	Cuboidal phloem sieve elements Strasburger cells	Jensen et al., 2012
	Companion cells	Strasburger cells	
Wood parenchymal fraction	High	Low	Nardini et al., 2011; Meinzer and McCulloh, 2013
Woody-tissue NSC content	High	Low	Hoch et al., 2003; Michelot et al., 2012
Wood density	High	Low	Poorter et al., 2012
Xylem embolism recovery capacity	High	Low	Bucci et al., 2003; Salleo et al., 2004; Brodribb et al., 2010
Sensitivity to freeze-thaw embolism	High	Low or absent	Cavender-Bares et al., 2005
Hydraulic safety margins	Narrow or negative	Wide	Choat et al., 2012
Water potential causing 50% loss of hydraulic conductivity	Low	High	Choat et al., 2012
Xylem capacitance	High (ring-porous)	Low	Meinzer and McCulloh, 2013
	Medium (diffuse-porous)		
Rate of C transfer	High	Low	Jensen et al., 2012
Sap flow velocity	High	Low	Jensen et al., 2012
Phloem conductivity	High	Low	Jensen et al., 2012
Phloem sieve-element resistance	Low	High	Jensen et al., 2012
Leaf lifespan	Shorter	Longer	Lusk et al., 2003
Shade tolerance	High	Low	Poorter et al., 2012
Interspecific shade-tolerance/drought-tolerance trade-off	Yes	Yes	Niinemets and Valladares, 2006
Mesophyllic conductance	High	Low	Niinemets et al., 2011
Photosynthetic capacity	High	Low	Lusk et al., 2003; Flexas et al., 2012
Stomatal density	High	Low	Flexas et al., 2012
Stomatal conductance sensitivity to VPD (m)	High (ring-porous)	Low	Johnson et al., 2012; Barbeta et al., 2013; Meinzer et al., 2013; Poyatos et al., 2013
	Medium-low (diffuse-porous)		
Distal leaf and root embolism and refilling	Rare	Frequent	Johnson et al., 2012

In support of these trends, Choat et al. (2012) recently reported that species in coniferous forests generally have a higher resistance to drought-induced cavitation and operate with wider hydraulic safety margins than do angiosperms. The minimum xylem pressures in conifers measured in the field were more positive than the xylem pressures causing a 50% loss of hydraulic conductivity, and thus the risk of hydraulic failure by collapse of the water-conducting system was low. In contrast, the hydraulic safety margins reported for angiosperms were narrower, being slightly positive or even negative.

The reported differences in stomatal sensitivity and hydraulic safety margins have in turn been functionally associated with different responses between both groups in the capacity of xylems to recover from embolisms. Recent studies have reported higher capacities in species with narrow safety margins and higher stomatal sensitivities to VPD (see Johnson et al., 2012 for a precise definition of stomatal sensitivity to VPD; Meinzer et al., 2013). The reversal of cavitation has been demonstrated to be feasible on an hourly or daily basis and to occur even under high xylem tension (Hacke and Sperry, 2003; Salleo et al., 2004; Brodersen et al., 2010; Zufferey et al., 2011). Two general but contrasting hydraulic strategies arise: (i) high cavitation resistance, low stomatal sensitivity to VPD and low resilience (gymnosperms) and (ii) low cavitation resistance but high resilience (angiosperms).

These two basic strategies are in turn functionally linked to anatomical differences in cell anatomy, NSC content, wood parenchymal fraction, and wood density (Table 2). For example, both the percentage of living parenchyma and the concentration of NSCs in the xylem are significantly higher in angiosperms than in conifers (Johnson et al., 2012 and citations therein). During the reversal of embolisms, vessel refilling probably requires an input of energy (Meinzer et al., 2013) and the mobilization of stored carbohydrates. Living wood parenchyma thus acts as a reservoir of both water and carbohydrates. Hence, NSCs stored in cells surrounding vessels are likely to be the source of sugars needed for the maintenance of vascular integrity (Brodersen et al., 2010; Sala et al., 2012). Sugars are possibly transferred from parenchymal cells to embolized vessels for establishing a gradient to drive water away from either the phloem (Nardini et al., 2011) or non-embolized vessels (Brodersen et al., 2010). Furthermore, Améglio et al. (2004) reported the catabolism of starch into sugars and the subsequent efflux from parenchymal cells to the vessels in late winter during the recovery of *Juglans regia* from cavitation induced by the winter freeze-thaw. Likewise, the reported differences between the capacities to reverse embolisms in angiosperms and conifers (Johnson et al., 2012; Brodersen and McElrone, 2013; Meinzer et al., 2013) are likely associated with the differences in sapwood NSC content between these two groups reported by Hoch et al. (2003). This empirical evidence suggests that NSC reserves in wood parenchymal cells play a key role in determining the hydraulic strategies of plants, because species with high NSC and parenchymal fractions would have a higher resilience to cavitation and thus could withstand a certain loss of hydraulic conductivity.

Finally, conifers and angiosperms also differ in cell anatomy and wood density (Table 2), and several studies suggest functional implications for these traits in climate-induced responses. For example, wood density has been proposed as a good predictor of the resistance of the xylem to drought stress, because species with denser wood tend to have a higher resistance to cavitation (Jacobsen et al., 2007; Pratt et al., 2007). Moreover, Ogasa et al. (2013) found a negative correlation between wood density and xylem recovery in deciduous angiosperm trees (*Salix*, *Betula, Carpinus, Cerasus*), suggesting in turn a negative association between increased cavitation resistance and resilience of xylem function. Wood density in Mediterranean evergreen shrubs was also negatively correlated with the percentage of parenchymal area in the xylem (Jacobsen et al., 2007). This correlation is consistent with the higher capacity of xylems to recover in species with wood of lower density reported by Ogasa et al. (2013), because living xylem parenchyma may be involved in the reversal of embolisms (Bucci et al., 2003; Brodersen et al., 2010; Nardini et al., 2011; Zufferey et al., 2011; Brodersen and McElrone, 2013). In addition, low wood density has been associated with high capacitance (Pratt et al., 2007; Sperry et al., 2008; McCulloh et al., 2012). In water-stressed plants, a higher capacitance facilitates the transient release of water stored in living wood cells to the conduit lumen, increasing xylem water potential (Meinzer et al., 2009; Barnard et al., 2011; Zhang et al., 2011).

The higher resistance of conifers to both freeze-thaw and drought-induced cavitation (Sperry and Sullivan, 1992; Wang et al., 1992; Choat et al., 2012) has also been associated with differences in wood anatomy (Table 2). The main difference in wood anatomy between angiosperms and gymnosperms is that the latter have tracheids that also provide mechanical strength (Hacke et al., 2001; Poorter et al., 2012). In particular, thick conduit walls providing mechanical strength have been suggested as the factor limiting the size of tracheids in conifers (Pittermann et al., 2006). Small tracheids are less prone to freeze-thaw cavitation in conifers (Tyree and Zimmermann, 1988; Sperry and Sullivan, 1992; Pittermann and Sperry, 2003), as are small vessels in angiosperms (Sperry and Sullivan, 1992), in which other woody cells such as fibers are responsible for mechanical support of the plant. In both groups, however, no direct relationship has been found between conduit size and drought-induced cavitation across species. Pit membrane area, though, must be limited (as it is where air-seeding develops) to achieve a certain level of safety from drought-induced cavitation, which in turn limits the surface area and thus the size of conduit cells (Hacke et al., 2006; Jansen et al., 2009; Brodribb et al., 2012).

We hypothesize that the reported trait differences between conifers and angiosperms (Table 2) constitute two different strategies that may imply qualitatively different growth responses to increased temperatures and drought in the Mediterranean region. The different stomatal responses to heat waves and summer droughts, inducing drought-avoidance strategies and stomatal closure in conifers, would be key to determining these different growth responses (Martínez-Ferri et al., 2000; Coll et al., 2013; Poyatos et al., 2013). Critically, the higher sensitivity of the stomatal conductance to increases in VPD in conifers may promote near-zero assimilation rates and may strongly limit carbon uptake and photosynthesis over extended periods (Martínez-Ferri et al., 2000; Johnson et al., 2012; Meinzer et al., 2013; Poyatos et al., 2013). Summer drought may strongly affect carbon dynamics and NSC mobilization and consumption in both conifers and angiosperms, for example by enhancing the catabolism of starch to soluble sugars for increasing xylem tension and sap osmolarity (Sala et al., 2012), mobilizing NSCs for embolism repair, producing soluble sugars to stabilize cellular proteins and membranes, stopping cell division and tree growth favoring in turn the accumulation of photosynthates in starch (Peñuelas and Estiarte, 1998; Estiarte and Peñuelas, 1999; Körner, 2003) or promoting increased allocation of NSCs in roots and declines in fine-root biomass (Anderegg, 2012). Even though the coupled effect of these complex processes on the carbon balance of the tree may be quite variable (species and site specific), we suggest that early stomatal closure and the associated larger reductions of assimilation rates in conifers may consistently produce a more negative impact on both carbon balance and growth responses of trees.

On the other hand, increased winter temperatures can reduce the costs associated with the impacts of freeze-thaw embolism and may also differently affect the carbon balance of angiosperms and conifers. Critically, angiosperms have a higher sensitivity to freeze-thaw embolism (Table 2) and may experience higher costs. This group could thus benefit more from increased winter temperatures. Higher winter temperatures would thereby entail fewer freeze-thaw cavitations, which are responsible for the almost complete loss of hydraulic conductivity in ring-porous species and for the partial loss in diffuse-porous species by late winter (Sperry and Sullivan, 1992). The restoration of water transport in angiosperms is achieved by the production of earlywood or by vessel refilling, which have carbon demands supplied by NSCs (Barbaroux and Bréda, 2002; Epron et al., 2012; Michelot et al., 2012). In contrast, since the xylems of conifers are highly resistant to freeze-thaw cavitation (Sperry and Sullivan, 1992; Brodribb et al., 2012), this group may not have very different NSC costs for the restoration of water transport after mild or cold winters.

Winter temperature is a major driver for switching carbon allocation either to storage or to growth and respiration (Epron et al., 2012; Körner, 2013) and for the conditioning accumulation of starch (Oleksyn et al., 2000). When temperature is too low for growth, carbon assimilation is still significant, so NSCs derived from winter photosynthesis are mainly allocated to storage during cold periods (Rossi et al., 2008; Fajardo et al., 2012). In addition, the catabolism of starch into soluble carbohydrates during cold periods may possibly maintain intracellular osmotic concentration, which is positively correlated with cold hardiness (Cavender-Bares et al., 2005; Morin et al., 2007). In both conifers and angiosperms, increased winter temperatures are likely to alter cambium activation, growth allocation and the dynamic balance among winter photosynthesis, starch storage, and soluble sugar concentrations.

Finally, increased winter, spring and autumn temperatures can significantly influence phenological responses, advancing winter cambium activation, spring bud burst and leaf unfolding or delaying autumn leaf fall (Peñuelas and Filella, 2001). The derived extension of the phenological period could have strong effects on tree height and growth (Vitasse et al., 2009a,b, 2013; Lenz et al., 2012). Both the phenological cycles and the growth-associated carbon dynamics, however, are qualitatively different in conifers, ring-porous deciduous trees, diffuse-porous deciduous trees, and evergreen oaks (Epron et al., 2012; Table 3). These differences suggest that these groups may qualitatively differ in the relative effects of increased spring temperatures on carbon dynamics and tree growth. For example, an increase in temperature early in the growing season may also increase vessel diameter in deciduous angiosperms but not in conifers (Matisons and Brumelis, 2012).

**Table 3:** **A brief summary of the seasonal dynamics of NSCs and growth phenology in deciduous broadleaf, evergreen broadleaf and coniferous trees**.

	**Winter**	**Spring**	**Summer**	**Autumn**
Deciduous angiosperm trees	Loss of hydraulic conductivity due to freeze-thaws, being higher in ring-porous than in diffuse-porous species (Sperry and Sullivan, 1992; Wang et al., 1992; Cavender-Bares et al., 2005; Michelot et al., 2012).	The onset of radial growth occurs before bud burst in ring-porous species and after bud burst in diffuse-porous species (Michelot et al., 2012).	NSCs in leaves decrease from summer through autumn (Hoch et al., 2003).	Allocation of carbon to storage (Epron et al., 2012).
	Before bud burst, some species may refill embolized vessels using NSCs (Améglio et al., 2004).	NSCs contribute to growth in both ring- and diffuse-porous species (Epron et al., 2012) but more importantly in ring-porous species (Barbaroux and Bréda, 2002; Palacio et al., 2011; Michelot et al., 2012).	The soluble fraction of NSCs is used to maintain xylem and phloem integrity and cell turgor under drought conditions (Sala et al., 2012). The soluble fraction increases in diffuse-porous species (Michelot et al., 2012). Another study, though, did not observe an increase in soluble fractions or observed reductions (Hoch et al., 2003).	Extended growing season (Peñuelas et al., 2002; Vitasse et al., 2009a,b; Gordo and Sanz, 2010).
		Starch content decreases in ring-porous trees, and sugars decrease in diffuse-porous trees (Michelot et al., 2012).	Higher stomatal conductance and dynamic embolism repair capacity may allow C assimilation even under a certain degree of water deficit (Johnson et al., 2012).	An increase of drought-induced embolism may also lead to premature leaf abscission (Wang et al., 1992).
		Milder winter temperatures may favor the formation of wider vessels in ring-porous species in early spring (Matisons and Brumelis, 2012).		
		Extended growing season with higher spring temperatures (Peñuelas et al., 2002; Gordo and Sanz, 2010).		
Evergreen angiosperm trees	Reduced losses in hydraulic conductivity caused by freeze-thaws, although evergreen trees are more resistant than deciduous species (Cavender-Bares et al., 2005).	Decline in NSC content by late spring (Rosas et al., 2013), probably invested in growth.	NSCs in leaves decrease from summer through autumn (Hoch et al., 2003).	Allocation of carbon to storage (Epron et al., 2012; Rosas et al., 2013).
	C assimilation allocated mainly to storage when temperature is too low for growth (Körner, 2003).	As in deciduous trees, vessel diameter is also constrained by winter temperatures (Cavender-Bares et al., 2005).	The soluble fraction of NSCs is used to maintain xylem and phloem integrity and cell turgor under drought conditions (Sala et al., 2012). The soluble fraction peaks in summer in some species (Rosas et al., 2013).	Mediterranean evergreens sometimes have a growth peak in autumn (Gutiérrez et al., 2011).
	NSC reserves increase throughout the winter (Rosas et al., 2013).	Extended growing season with higher temperatures (Peñuelas et al., 2002; Gordo and Sanz, 2010).	Do not close stomata completely even under high evaporative demand and low soil water content (Ogaya and Peñuelas, 2003; Barbeta et al., 2012).	
	Annual peak in photosynthetic rates for some species (Ogaya and Peñuelas, 2003).		Narrower xylem vessels than in deciduous oaks reduce losses of hydraulic conductance (Sperry and Sullivan, 1992; Wang et al., 1992 in other species).	
Conifers	Freeze-thaw resistant species. No accumulated losses in hydraulic conductivity (Wang et al., 1992).	Carbohydrate demand of new-leaf cohorts is supplied mainly by older cohorts (Eilmann et al., 2010; Michelot et al., 2012).	NSCs in leaves decrease from summer through autumn (Hoch et al., 2003).	Mediterranean conifers have a growth peak in autumn (Camarero et al., 2010; Pasho et al., 2012).
	Low temperatures may result in an increase of NSCs (Hoch, 2008; Fajardo et al., 2012; Gruber et al., 2012; Hoch and Körner, 2012).	Growth is apparently not dependent on NSCs (Michelot et al., 2012).	Peak of starch content before the onset of latewood (Oberhuber et al., 2011).	Allocation of carbon to storage (Epron et al., 2012).
	High minimum temperatures may advance earlywood formation in Mediterranean conifers (Pasho et al., 2012).	High temperatures may lead to an earlier onset of radial growth (Camarero et al., 2010).	Xylem structure is in general highly resistant to cavitation (Choat et al., 2012; Johnson et al., 2012).	
			Very tight stomatal control may lead to near-zero carbon assimilation (Poyatos et al., 2013).	

### Phenology (Hypothesis 1.2)

An average lengthening of the growing season of about 11 days has been detected in Europe from the early 1960s to the end of the twentieth century (Menzel and Fabian, 1999; Peñuelas and Filella, 2001; Linderholm, 2006; Menzel et al., 2006). Growing season length has a strong effect on tree productivity, Consequently, the reported temperature-induced changes in phenology could affect tree growth responses (White et al., 1999; Kramer et al., 2000; Picard et al., 2005; Delpierre et al., 2009; Richardson et al., 2009; Vitasse et al., 2009a,b; Dragoni et al., 2011; Rossi et al., 2011; Lugo et al., 2012). Empirical evidence in temperate trees suggests that the productivity of evergreen needleleaf forests is less sensitive to phenology than is productivity of deciduous broadleaf forests (Welp et al., 2007; Delpierre et al., 2009; Richardson et al., 2010). For instance, Churkina et al. (2005) reported a different sensitivity of net ecosystem productivity to growing season length in deciduous forests (5.8 + 0.7 g C m^−2^ d^−1^), compared with evergreen needleleaf forests (3.4 + 0.3 g C m^−2^ d^−1^). Similarly, Piao et al. (2007) reported different sensitivities of gross ecosystem productivity to growing season length (9.8 + 2.6 g C m^−2^ d^−1^ in deciduous forests, compared with 4.9 + 2.5 g C m^−2^ d^−1^ in evergreen needleleaf forests). To our knowledge, it remains untested whether qualitatively different phenology responses in Mediterranean conifers and angiosperm trees may occur and translate into different tree growth responses on a large scale.

However, other evidence points to complex and species-specific effects of phenology on tree growth. For instance, for both conifer and angiosperm trees, a variety of species-specific responses in bud burst and bud set have been reported along altitudinal and latitudinal gradients, reporting both advances, delays and non-significant clines (Vitasse et al., 2009a,b, 2013; Alberto et al., 2013). For example, depending on the species considered, Vitasse et al. (2009b) found positive and negative correlations between advanced leaf emergence and annual growth. Moreover, warming can produce complex and counter-intuitive effects on phenology and growth. For example, strong warming in winter could slow the fulfillment of chilling requirements, which may delay spring phenology and growth (Körner and Basler, 2010; Yu et al., 2010) and affect differently early and late successional species (Körner and Basler, 2010).

In the Mediterranean region, mean annual and maximum temperatures have been identified as the major drivers of deciduous tree phenology (Gordo and Sanz, 2010). However, the effects of temperature on the phenology of many conifer and angiosperm tree species in the Mediterranean basin remain yet relatively poorly quantified (Maseyk et al., 2008). It remains also uncertain whether trade-offs between the advance of spring flushing date and the increased risk of frost damage may differ qualitatively between Mediterranean trees (Lockhart, 1983; Lechowicz, 1984). The same applies for trade-offs between delayed autumn leaf fall date, increased autumn photosyntate storage, and increased late-autumn frost damage risk and incomplete leaf nutrient remobilization costs (Lim et al., 2007). Finally, in the Mediterranean basin, drought periods significantly affect both leaf phenology and tree growth in both conifer and angiosperm trees (de Luis et al., 2007, 2011; Camarero et al., 2010). For instance, increased leaf retention rate and lifespan have been observed in response to drought in holm oak forests (Bussotti et al., 2003; Misson et al., 2010). Drought also causes foliage to fall earlier and results in incomplete leaf nutrient translocation and increased nutrient concentration in litter (Martínez-Alonso et al., 2007).

### Intra-specific competition, inter-specific competition and forest succession (Hypothesis 1.3)

Empirical studies reveal that intra-specific competition acts as a major determinant of growth patterns in Mediterranean forests in both conifer and angiosperm trees (Gómez-Aparicio et al., 2011). Forest densification due to land abandonment and the advance of succession is occurring over extensive areas, increasing competition, reducing tree growth, and increasing mortality (Gómez-Aparicio et al., 2011; Vilà-Cabrera et al., 2011; Coll et al., 2013). Coll et al., (2013) reported much higher negative effects of forest stand basal area on conifer growth than in angiosperm trees in both dry and wet extremes of a large-scale rainfall gradient, and these trends were paralleled by higher effects of basal area on small-tree mortality observed in conifers. These results coincide with studies revealing oaks less sensitive to competition than pines in this area (Sánchez-Gómez et al., 2008; Gómez-Aparicio et al., 2011).

Inter-specific competition also plays an important role in determining growth responses in Mediterranean conifer and angiosperm trees. Specifically, large-scale surveys suggest that small-sized conifers are more sensitive to growth suppression by late successional species (Gómez-Aparicio et al., 2011; Zavala et al., 2011; Coll et al., 2013). Angiosperm trees are significantly expanding their distributional ranges, increasing recruitment across extensive areas (Coll et al., 2013; Vayreda et al., 2013). Morover, during the last decades the expansion of the dominant angiosperm tree *Quercus ilex* has negatively influenced the recruitment success of five *Pinus* species on a large scale in this area (Carnicer et al., 2013a).

### Size, age, and allometry (Hypothesis 1.4)

Mediterranean conifers differ from angiosperm trees in their allometrical relationships between tree size (diameter at breast height) and crown growth variables (Poorter et al., 2012). The peak of crown growth is generally reached at lower sizes in conifers, which also show a much steeper decrease with size than broadleaved species (Poorter et al., 2012). These different allometric relationships are in turn associated with several other traits (maximal height, crown size, shade tolerance, wood density, apical dominance) and also interact with local habitat aridity (Poorter et al., 2012). Similarly, Gómez-Aparicio et al. (2011) reported that in Iberian forests competitive effects for conifers scale approximately quadratically with diameter at breast height (dbh^2^) and linearly for broadleaved trees. To our knowledge, it remains untested whether these different allometric relationships might be related to the contrasting tree growth responses to temperature reported in Mediterranean conifers and angiosperm trees (Gómez-Aparicio et al., 2011).

### Drought and temperature (Hypothesis 1.5)

Large-scale studies demonstrate that drought and increased temperatures significantly limit tree growth in xeric regions of the Mediterranean basin (Andreu et al., 2007; Martínez-Alonso et al., 2007; Sarris et al., 2007; Bogino and Bravo, 2008; Martínez-Vilalta et al., 2008; Gómez-Aparicio et al., 2011; Vilà-Cabrera et al., 2011; Candel-Pérez et al., 2012; Sánchez-Salguero et al., 2012; Vayreda et al., 2012; Coll et al., 2013) and produce qualitatively different ecophysiological responses in Mediterranean conifers and angiosperm trees (Martínez-Ferri et al., 2000; Zweifel et al., 2007; Eilmann et al., 2009). For instance, while drought often results in early stomatal closure in Mediterranean conifers (Martínez-Ferri et al., 2000; Klein et al., 2011; Poyatos et al., 2013), angiosperm trees are able to maintain substantial transpiration levels during summer drought events (Quero et al., 2011).

Drought largely determines cambium growth in Mediterranean forests, producing plastic and seasonally variable patterns, ranging from one single annual peak to markedly bimodal trends (Maseyk et al., 2008; Camarero et al., 2010; de Luis et al., 2011). However, large-scale studies in the Iberian peninsula reveal that competition effects on growth are often stronger than drought effects (Gómez-Aparicio et al., 2011; Coll et al., 2013). Nevertheless, strong interactions between competition and drought effects have been reported, and significantly increase at the edge of climatic gradients (Linares et al., 2010; Vayreda et al., 2012; Coll et al., 2013; Ruiz-Benito et al., 2013). Finally, there is also some evidence of individual predispositions to winter-drought induced tree dieback in *P. sylvestris* (Voltas et al., 2013), local adaptation for water use efficiency in *P. halepensis* (Voltas et al., 2008), and correlations of temperature and genetic variability at candidate loci for drought tolerance in *P. halepensis* and *P. pinaster* (Grivet et al., 2011), suggesting important interactions between individual adaptive traits and drought impacts.

### Winter freezing (Hypothesis 1.6)

Angiosperm trees are more vulnerable to freeze-thaw embolism and this may contribute to explain the dominance of conifer trees at high altitudes (Cavender-Bares et al., 2005; Brodribb et al., 2012) and could in turn result in qualitatively different growth responses in conifers and angiosperm trees. For example, Gómez-Aparicio et al. (2011) reported that Atlantic deciduous broadleaved trees in the Iberian peninsula had lower competitive response ability at lower temperatures, in contrast to mountain conifer species. In this study, tree growth of Atlantic deciduous broadleaved trees was negatively affected by low temperatures (Gómez-Aparicio et al., 2011). In line with this, several studies have demonstrated that low winter temperatures directly inhibit cell division and tree growth in cold localities (Körner, 1998, 2013; Fajardo et al., 2012).

### Interactions between multiple factors (hypothesis 1.7)

Tree growth patterns in the Iberian peninsula have several contributing drivers that interact along geographical gradients (Coll et al., 2013). For instance, Gómez-Aparicio et al. (2011) studied tree growth responses in 15 tree species in Spain and reported that sensitivity to competition increased with decreasing precipitation in all species. Notably, the best predictive models for tree growth in Gómez-Aparicio et al. (2011) included interactions between size, competitive effects and climate variables. Similarly, Coll et al., (2013) modeled growth responses in the Iberian peninsula and reported a significant increase in the strength of interactions between tree size, tree height and climate variables at the drier and wetter edges of rainfall gradients. These interactions could increase with ongoing climate change, and several studies suggest that warming could increase competition for water in Mediterranean forests (Linares et al., 2010).

### Local adaptation, individual- and provenance variation (Hypothesis 1.8)

Local selection processes may affect the adaptive traits determining the different growth responses to temperature observed in Iberian conifers and angiosperm trees. For example, provenance studies in both conifer and angiosperm trees have revealed genetic differences in growth rates and other growth-related traits (age at reproduction, timing of bud burst and bud set, leaf traits, flowering phenology), suggesting that populations are often adapted to their local conditions of temperature and water availability (Rehfeldt, 1978, 1982, 1988; Borghetti et al., 1993; Climent et al., 2008; Mátyás et al., 2009; Rose et al., 2009; Ramírez-Valiente et al., 2010, 2011; Santos et al., 2010; Chmura et al., 2011; Robson et al., 2012; Alberto et al., 2013). In provenance trial studies, populations from cold environments often cease growth earlier, while populations from warm localities generally grow faster (Alberto et al., 2013). Notably, local selection for increased growth rates may induce lower resistance to drought and frost. For instance, in conifers fast-growing provenances often exhibit lower cold hardiness and/or lower resistance to drought stress (Hannerz et al., 1999; Cregg and Zhang, 2001; Chuine et al., 2006). These differences have been attributed to trade-offs between resistance to frost and drought and growth (Chuine et al., 2006 and see Martin St Paul et al., 2012).

### Phenotypic plasticity (Hypothesis 1.9)

Mediterranean trees show strong plastic responses in tree growth patterns, which are associated with seasonal climate variability (e.g., Camarero et al., 2010; de Luis et al., 2011). Critically, phenology and growth plasticity responses differ between provenances and species and may determine observed demographic and evolutionary responses to global warming (Nicotra et al., 2010). For example, low elevation provenances often exhibit greater phenological plasticity to temperature than high elevation provenances (Vitasse et al., 2013) and this could in turn influence observed tree growth responses. To our knowledge, it remains untested whether Mediterranean conifers exhibit higher growth plasticity than angiosperm trees, although it has been reported that Iberian conifers show higher growth rates than angiosperm trees in absence of competition (Gómez-Aparicio et al., 2011; Poorter et al., 2012).

### Experimental assessment of the relative contribution of the hypotheses

The available empirical evidence suggest that several factors interact and seem to determine contrasting growth responses to temperature in Mediterranean conifer and angiosperm trees. Therefore, improved experimental approaches are required to quantitatively assess the relative importance of these factors. While several experimental and observational approaches could be applied, we suggest that reciprocal provenance trial experiments may be especially suited for this purpose. Previous studies assert that multiple common garden experiments located in latitudinal and altitudinal gradients are particularly relevant to study phenology and growth responses to temperature (Reich and Oleksyn, 2008; Vitasse et al., 2010). Furthermore, the inclusion of different provenances in these reciprocal experiments allows the quantification of environmentally induced phenotypic plasticity, genotypic variance and their interaction (e.g., Vitasse et al., 2013). Complementarily, drought effects on growth could be studied by manipulative experiments combined with reciprocal common garden designs (reviewed in Klein et al., 2011; Wu et al., 2011). Similarly, the effects of intra- and inter-specific competition could be studied manipulating tree densities and composition in different experimental groups. Finally, to assess tree size effects and allometric relationships, the study of saplings of different ages would be required. Alternatively, long-term experiments could provide also relevant information to quantify allometric relationships. Finally, in all these experimental designs, the periodic measurement of ecophysiological traits should be implemented to assess their seasonal variation and their putative role in determining growth responses.

## Complex and multiple effects of temperature and drought on tree physiology

Climate produces multiple and complex effects on tree physiology. As highlighted in Table 1, we expect that multiple physiological processes can simultaneously react to the changes in environmental temperatures and influence growth responses. For example, temperature and drought directly affect several ecophysiological processes such as carbon and nutrient uptake, carbon allocation between tissues, photosynthesis, respiration, processes of embolism prevention and repair, phenological cycles, cambium reactivation, cell division and expansion or carbon transfer rates (Körner, 1998; Bréda et al., 2006; Rennenberg et al., 2006; Sanz-Pérez et al., 2009; Camarero et al., 2010; Epron et al., 2012; Michelot et al., 2012). Moreover, these direct climatic effects on tree physiology can in turn produce secondary indirect effects, for example the promotion of signaling and regulatory responses, acclimation and phenotypically plastic responses or changes in gene expression (reviewed in Peñuelas et al., 2013b). Table 4 provides a brief, non-exhaustive description of the diverse effects of temperature and drought on tree physiology. It is important to bear in mind that all these ecophysiological processes often have different sensitivities and thresholds to temperature and water deficit. For example, tree growth and cambium activation are more sensitive to low temperatures than is photosynthesis (Körner, 1998; Fajardo et al., 2012). In addition, as shown in Table 4, responses to climate are often species or tissue specific or depend on developmental stage and seasonal phase and can be influenced by regulatory feedbacks that can often imply multi-tissue coordinated responses. Despite the overwhelming complexity and diversity of the effects of temperature and drought reported in Table 4, several studies have demonstrated consistent differences between major plant groups, such as conifers and angiosperms, in climate-induced responses (e.g., Way and Oren, 2010; Gómez-Aparicio et al., 2011; Coll et al., 2013).

**Table 4:** **A non-exhaustive and synthetic review of the different effects of temperature (A) and drought (B) on different tree physiological processes**.

	**References**
**(A) EFFECTS OF TEMPERATURE ON TREE PHYSIOLOGY**	
*Photosynthesis*. Temperatures higher/lower than the optimum decrease photosynthesis and affect multiple biochemical processes. For example, high temperatures can reduce the efficiency of electron transport in the thylakoid membrane of chloroplasts, which in turn down-regulate the content of ribulose-1,5-bisphosphate and deactivate Rubisco. High temperatures also inhibit Rubisco activase, due to their low thermal optimum. The solubility of the two substrates of Rubisco, CO_2_, and O_2_, is differentially affected by temperature, stimulating photorespiration and inhibiting photosynthesis at high temperatures.	Rennenberg et al., 2006
	Morin et al., 2007
	Kattge and Knorr, 2007
	Chaves et al., 2012
	Flexas et al., 2012; Sharkey and Bernacchi, 2012
Photosystem II is also sensitive to high temperatures, which stimulate mechanisms to avoid photo-oxidation and membrane denaturation, such as isoprene production and the xanthophyll cycle.	
Low temperatures cause a variety of physiological and acclimative responses, including modifications in the structure of the thylakoid membrane in chloroplasts, alleviation of photoinhibition through upregulation of carbon metabolism and increased synthesis of storage carbohydrates, increased production of antioxidants, prevention of intracellular freezing by increased soluble carbohydrates (mobilization of starch to sucrose) and changes in gene expression and signaling pathways.	
The growth environment of plants determines the temperature optimum of photosynthesis. In warmer environments, plants acclimate to increase the thermal optimum of the maximum carboxylation velocity (Vcmax) and the maximum potential rate of electron transport (Jmax).	
Above the thermal optimum for photosynthesis, the emission of biogenic volatile organic compounds such as isoprene and monoterpenes progressively increases.	Llusià and Peñuelas, 2000; Rennenberg et al., 2006
*Leaf respiration* is strongly affected by temperature, increasing at high temperatures (e.g., above 35–40°C) and peaking at higher temperatures than photosynthesis.	Rennenberg et al., 2006; Smith and Dukes, 2013
High temperatures often increase net primary production and plant growth. In cold-adapted trees, photosynthesis is less sensitive to low temperatures than is tree growth (cell division and growth, cambium activation). In alpine treelines, new tissue formation is nearly absent at temperatures around 5°C, but considerable rates of photosynthesis are maintained between 0 and 10°C.	Körner, 1998; Way and Oren, 2010; Wu et al., 2011; Fajardo et al., 2012; Lenz et al., 2012
Higher temperatures influence foliar phenology, promoting earlier bud burst and delaying leaf fall.	Peñuelas and Filella, 2001; Peñuelas et al., 2002; Vitasse et al., 2009a,b, 2013
In the absence of drought, temperature often increases *nutrient-uptake* capacity (NH^+^_4_, NO^−^_3_, PO^−^_43_, K^+^). Temperature can also increase both xylem loading of amino compounds and nitrogen allocation in aboveground tissues.	Rennenberg et al., 2006
Freezing causes cell dehydration, formation of ice in intracellular spaces and embolism. Buds are more resistant than leaves to frost.	Morin et al., 2007; Augspurger, 2009
Temperature, in absence of drought, positively affects rates of soil respiration and litter decomposition.	Wu et al., 2011
Organs, individuals, life stages and species consistently differ in their phenological responses to temperature and sensitivity to damage from frost and drought.	Niinemets and Valladares, 2006; Morin et al., 2007; Augspurger, 2009
**(B) EFFECTS OF DROUGHT ON TREE PHYSIOLOGY**	
*Photosynthesis*. Drought limits photosynthesis by stomatal closure, diffusion limitations in the mesophyll and metabolic impairment. It can also limit photosynthesis via secondary effects, such as reduced hydraulic conductance and oxidative stress.	Chaves et al., 2012
	Sharkey and Bernacchi, 2012
Drought activates diverse signaling pathways associated with stomatal closure. For example, it modifies abscisic acid (ABA) signaling in leaves, shoots and roots; increases xylem-sap pH and changes aquaporin concentrations, leaf hydraulic conductance signals and electric signals.	
Drought reduces osmotic potential in the soil and predawn leaf water potentials and limits water uptake. To maintain water uptake, plants increase the production of osmolites, down-regulate electron flux and increase the activity of antioxidant enzymes. Drought can also increase the degradation of foliar proteins and the concentration of soluble amino acids and NSCs in the leaves, which may act in turn as osmoprotectants to stabilize proteins and membranes. Drought also promotes an increase in the concentrations of soluble antioxidants.	Rennenberg et al., 2006.
**(B) EFFECTS OF DROUGHT ON TREE PHYSIOLOGY**	
Severe water stress can produce irreversible or persistent damage in the photosynthetic apparatus of leaves (relative to leaf lifespan).	Sharkey and Bernacchi, 2012
Drought reduces tree growth, net primary production, cambium activity, cell division and growth.	Eilmann et al., 2009; Camarero et al., 2010; Wu et al., 2011; de Luis et al., 2011
Drought reduces C transfer rates.	Barthel et al., 2011; Epron et al., 2012
Drought is associated with acclimative responses such as mid-term reductions in total leaf area and defoliation.	Bréda et al., 2006; Ogayaand Peñluelas, 2006; Carnicer et al., 2011
Drought promotes an increase in NSCs in roots and a decrease in fine-root biomass.	Anderegg, 2012; Anderegg et al., 2012
Drought alters nutrient-uptake processes, for example promoting increases in ammonification and decreases in denitrification in the soil.	Gessler et al., 2005
Isoprenoid emissions can be negatively affected by drought stress and increase during plant recovery after drought.	Rennenberg et al., 2006; Peñuelas and Staudt, 2010
Drought can increase the accumulation of ethylene in shoots, in turn reducing shoot growth.	Chaves et al., 2012
Water deficit can reduce N uptake from the soil and change N partitioning between roots and shoots, increasing N content in the roots.	Rennenberg et al., 2006
Omic studies reveal that drought produces changes in *gene regulation*, for example promoting proline synthesis and down-regulating proline degradation.	Chaves et al., 2012; Peñuelas et al., 2013a
Negative effects of drought differ between phases of plant development and annual phenophases and are usually stronger during reproductive and leaf-emergence phases in deciduous trees.	Chaves et al., 2012
Drought produces tissue-specific signaling responses in roots, shoots and leaves and tissue-specific interactions between signaling factors. For example, different interactions between ABA and ethylene have been reported in roots and shoots.	

## Empirical patterns in the Iberian peninsula: the negative synergistic effects of increased temperatures and forest successional advance

**Figure 1:**
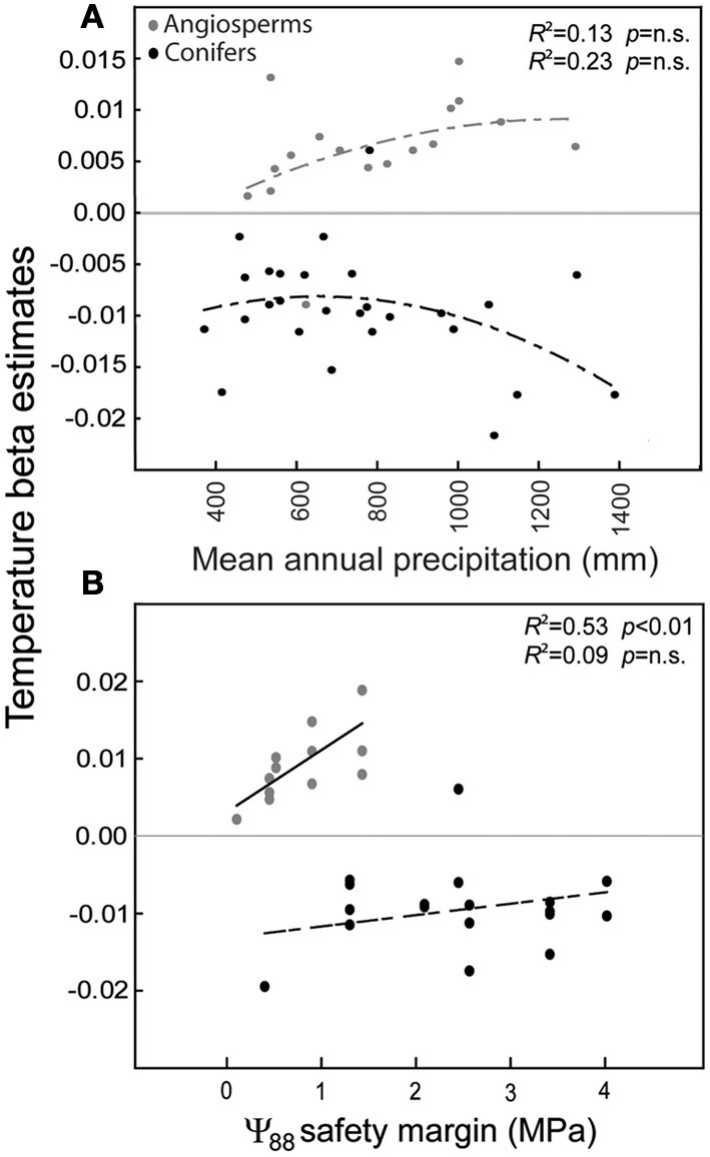
**Summary of the variation in the effect of temperature on tree growth along a rainfall gradient (A) and across interspecific differences in hydraulic safety margins (B) in conifers (black dots) and angiosperms (gray dots)**. The tree species included in the analysis are: *Fagus sylvatica, Quercus ilex, Q. pubescens, Q. pyrenaica, Q. robur, Abies alba, Pinus halepensis, P. nigra, P. pinaster, P. pinea, P. sylvestris*, and *P. uncinata. P. radiata* and *Q. suber* were only included in panel **(A)** due to a lack of data for hydraulic safety margins. Coll et al., (2013) applied generalized linear models (GLM) to study tree growth responses (dependent variable) and assessed the following independent predictors: (i) climate and topography (Emberger water deficit index, mean annual temperature, terrain slope), (ii) forest stand structure (tree density, basal area), (iii) soil (organic layer depth), (iv) individual tree traits [tree height, diameter at breast height (DBH)], and (v) management practices (e.g., plantations). Beta estimates in panels **(A)** and **(B)** show the reported significant effects of temperature on tree growth in GLM analyses (Coll et al., 2013). n.s. means not significant.

**Figure 2:**
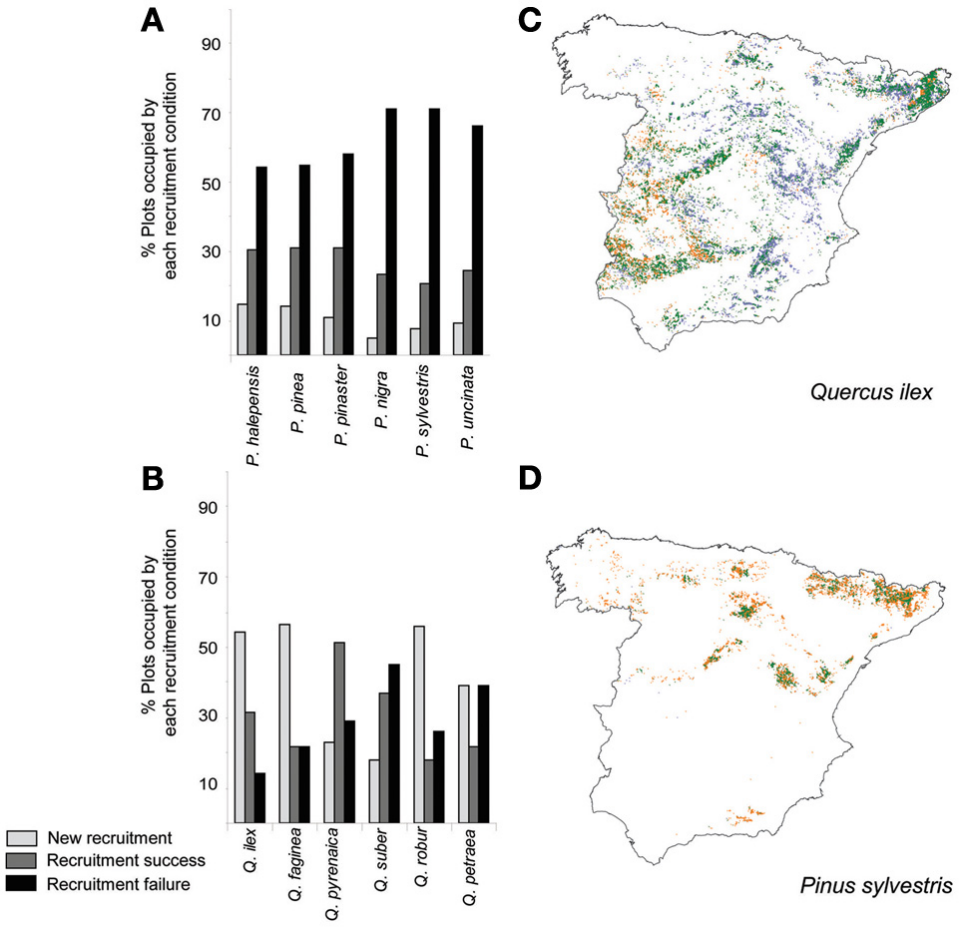
**Contrasting large-scale trends in tree recruitment observed in the Iberian peninsula for small saplings (height <30 cm) in Conifers (*Pinus*) and Angiosperm trees (*Quercus*). (A)** Variation in the percentage of plots with recruitment success (gray), recruitment failure (black) and new recruitment areas (plots without adult trees of the focal species in which small recruits or resprouts were detected) in *Pinus* species; **(B)** Variation in the percentage of plots with recruitment success (gray), recruitment failure (black) and new recruitment areas in *Quercus* species. **(C)** Spatial trends in recruitment for the dominant species *Quercus ilex*. Blue areas indicate new recruitment areas (i.e., areas with recruits but absence of adult trees), orange areas illustrate recruitment failure and green areas illustrate recruitment success (i.e., areas characterized by the presence of both adult and small saplings). **(D)** Spatial trends in recruitment for *Pinus sylvestris*. Differences between recruitment trends in *Pinus* and *Quercus* were significant (see Carnicer et al., 2013a for a detailed statistical test. Average proportion of plots with recruitment failure: *F* = 16.64, *P* = 0.002; average proportion of plots with new recruitment: *F* = 35.04, *P* = 0.0001). Data were obtained from the Spanish National Forest Inventory, consisting in a regular grid of circular plots at a density of 1 plot/km^2^.

In the Mediterranean basin, land use changes often negatively interact with increased temperatures and drought events and result, in diverse taxonomic groups, in negative demographic trends detectable on a large scale (Linares et al., 2010; Stefanescu et al., 2011; Carnicer et al., 2013b). In the case of Iberian forests, increased stand competition due to forest successional advance and forest densification has been identified as a major driver of tree demographic responses (Gómez-Aparicio et al., 2011; Carnicer et al., 2013a). Notably, stand competition interacts with temperature and drought responses in this region, especially in the drier and wetter edges of rainfall gradients (Linares et al., 2010; Coll et al., 2013). In this section we briefly review the contrasting demographic trends to temperature observed in Conifers and Angiosperm trees in the Iberian peninsula. Forest succession is currently favoring a shift toward an increased dominance of angiosperm trees on a large scale (Carnicer et al., 2013a; Coll et al., 2013; Vayreda et al., 2013). On top of this, recent studies (Gómez-Aparicio et al., 2011; Coll et al., 2013) show that tree growth responses to temperature differ between conifers and angiosperms on a large scale in the Mediterranean forests of the Iberian Peninsula. Large-scale empirical patterns of the responses of tree growth to temperature along a gradient of rainfall in Spain are illustrated in Figure 1A, showing contrasting responses in conifers (black dots) and angiosperms (gray dots). Panel **(A)** depicts the variation of temperature beta estimates on species-specific responses of tree growth in forests located along a gradient of rainfall (Coll et al., 2013). Tree-growth data were obtained from the Spanish National Forest Inventory, which comprises a wide range of forest types, from typically Mediterranean lowland stands to northern temperate forests with strong Atlantic influences to alpine forests located in the Pyrenees (Coll et al., 2013). To analyze the relationship between growth responses to temperature and trait differences between conifers and angiosperms, we used hydraulic safety margins as a key synthetic variable of the hydraulic strategy of each species (Figure 1B). Panel **(B)** depicts two separate linear regressions between the temperature beta estimates on growth and the species-specific hydraulic safety margins. Hydraulic safety margins were obtained from Cochard and Tyree (1990), Cochard (1992, 2006), Tognetti et al. (1998), Cochard et al. (1999), Martínez-Vilalta and Piñol (2002), Martínez-Vilalta et al. (2002, 2009), Oliveras et al. (2003), Corcuera et al. (2006), and Choat et al. (2012). A significant linear relationship between growth responses to temperature and species-specific hydraulic safety margins was only observed in angiosperms (Figure 1B), and conifers had significantly larger hydraulic safety margins (Figure 1B). Across the studied range of hydraulic safety margins, the temperature beta estimates were positive for angiosperms (gray dots) but negative for conifers (black dots), regardless of mean precipitation (Figure 1A). This result is consistent with those of other studies on the effects of climate in the Iberian Peninsula reporting negative significant effects of temperature on tree growth in conifers (Gómez-Aparicio et al., 2011; Candel-Pérez et al., 2012; Büntgen et al., 2013). Figure 2 illustrates the specific forest successional context in which the reported contrasting effects of temperature on tree growth previously reported occur. Conifers show a significantly higher percentage of plots characterized by recruitment failure (Figure 2A; Carnicer et al., 2013a). In contrast, *Quercus* species showed a much larger percentage of recently colonized plots and/or resprouting areas (i.e., plots without adult trees but in which recruits and/or resprouts of the focal species were detected, Figure 2B, Carnicer et al., 2013a). Overall Figures 1, 2 suggest that in this area the negative effects of warming and forest successional advance could synergistically impact conifer species during the next decades.

## Discussion

We have reviewed the different hypotheses that may contribute to explain the recently reported different growth responses to temperature in Mediterranean angiosperm and conifer trees (Table 1; Gómez-Aparicio et al., 2011; Coll et al., 2013). Conifer and angiosperm trees differ in the effects of phenology on tree productivity, in their sensitivity to stand competition and in their growth allometry. In addition, they consistently differ in an integrated suite of key traits, including different hydraulic safety margins, stomatal sensitivity, embolism repair capacity and xylem anatomy, suggesting two contrasting ecophysiological strategies to confront drought and extreme temperature events. However, for many Mediterranean conifer and angiosperm trees, detailed empirical studies contrasting the relative effect on tree growth of the factors listed in Table 1 are still lacking. For example, it is not clear whether temperature-induced shifts in phenology consistently differ between conifers and angiosperm trees in the Mediterranean region and how these shifts in phenology could differentially alter their productivity. Similarly, the seasonal dynamics of key traits, like cambium growth, tissue NSC content or sap flow, remain yet poorly quantified for many species. So it is clear that improved experimental approaches to contrast and assess the relative effect of the reviewed hypotheses are required (Table 1) if we are to explain the contrasting growth trends reported in recent large-scale studies in these two groups (Gómez-Aparicio et al., 2011; Coll et al., 2013; Figure 1). We have suggested that the relative effects of these factors (Table 1) could be contrasted in reciprocal common garden experiments located in altitudinal or latitudinal gradients that provide an ideal design to estimate temperature effects on phenology and growth, and also allow the estimation of local adaptation and phenotypic plasticity (Vitasse et al., 2009a,b, 2013). In these reciprocal transplant experiments, detailed quantitative analysis of the relationships between growth measures and hydraulic safety margins, stomatal sensitivities to VPD, embolism repair activity and NSC carbon dynamics in wood parenchyma and other tissues would be ideally required to clarify the relative importance of these processes and their dynamic inter-relationships (Camarero et al., 2010; de Luis et al., 2011; Oberhuber et al., 2011; Michelot et al., 2012; Pasho et al., 2012).

The available empirical evidence (Gómez-Aparicio et al., 2011; Coll et al., 2013; Figure 2) suggests that increased stand competition associated with successional advance is a primary driver of growth trends in the forests of the Iberian peninsula. So it would be key to simulate this factor in the proposed transplant experiments, manipulating sapling densities and composition. We suggest that mixed pine-oak designs would be especially interesting because recent studies describe the widespread expansion of *Quercus* saplings and resprouts in the Iberian peninsula and limited recruitment in *Pinus* species (Carnicer et al., 2013a; Coll et al., 2013; Vayreda et al., 2013; Figure 2). Moreover, *Quercus ilex* seems to act as a keystone species in driving these limited recruitment trends, inhibiting recruitment in five different *Pinus* species (Rouget et al., 2001; Carnicer et al., 2013a). In addition, several studies report that pines are more sensitive to competition and their growth can be largely suppressed with the advance of succession, specially on sapling and young stages (e.g., Gómez-Aparicio et al., 2011; Zavala et al., 2011; Coll et al., 2013). Therefore, these processes should be ideally considered in reciprocal transplant experiments, to allow the experimental study of the combined negative synergistic effects of warming and increased successional advance.

Ideally, the experimental approaches tested in these common garden experiments should simulate future forest scenarios in the face of climate change in the Iberian Peninsula. However, future scenarios in this region remain uncertain. For example, the available model predictions vary from important range contractions to substantial range expansions (Benito Garzón et al., 2011; Keenan et al., 2011; Ruiz-Labourdette et al., 2012; García-Valdés et al., 2013). We have suggested a possible scenario of global change dominated by the widespread expansion of angiosperm broadleaved trees, increased suppression of pine growth and recruitment by *Q. ilex* and specially acute negative demographic trends in mountain pines (*Pinus sylvestris*, *Pinus nigra* and, to a less extent, *P. uncinata*) (Figure 2; Carnicer et al., 2013a). Other major uncertainties in future forest scenarios are related to non-linear dynamics in fire activity (Loepfe et al., 2012), changes in fire-climate relationships motivated by the generalized advance of forest succession and the expansion of *Quercus* species that may substantially alter the distribution of forest fuel over extensive areas (Pausas and Paula, 2012; Carnicer et al., 2013a), and the future changes in land uses induced by shifts in global energy policies and the increased use of forests as a local energy source (Peñuelas and Carnicer, 2010, Carnicer and Peñuelas, 2012).

In Table 3 we have also discussed how tree carbon dynamics may be interacting with climate-induced responses in the seasonal variation of photosynthesis, annual growth cycles, embolism prevention, embolism repair and refilling and stomatal responses. Important gaps in our knowledge remain, and we lack a clear picture of how tissue-specific NSC concentrations vary seasonally, their interspecific variation and how these seasonal variations are connected to the diverse physiological functions examined (i.e., carbon buffer function, winter- and drought-induced embolism repair, embolism prevention, bud burst and leaf unfolding, responses of root and stem growth and respiration) (Hoch et al., 2003; Epron et al., 2012; Michelot et al., 2012; Sala et al., 2012). Another aspect that merits more attention in future empirical tests is the putative existence of compensatory dynamics across seasons in the effects of climate on tree physiology. For example, higher temperatures may reduce the costs of winter embolism in broadleaved deciduous trees, lengthen the growing season or increase the production of photosynthates in spring. These changes could in turn allow higher NSC storage in spring, which could increase embolism repair capacity during summer droughts (compensatory seasonal effects).

In summary, a review of the existing empirical evidence suggests that contrasting demographic responses in Mediterranean conifer and angiosperm trees are currently occurring, due to both widespread forest successional advance and to divergent growth responses to temperature. Trait-based differences in these two groups may contribute to explain their different responses to temperature (Table 2, Figure 1) and their different role during successional processes in this region (Figure 2, Table 2, reviewed in Zavala et al., 2011; Poorter et al., 2012; Sheffer, 2012). Reciprocal common garden experiments may offer a very promising tool to develop integrative tests of the diverse factors reviewed (Table 1) and to simulate the synergistic negative effects of forest successional advance and climate warming on conifer species (Carnicer et al., 2013a).

### Conflict of interest statement

The authors declare that the research was conducted in the absence of any commercial or financial relationships that could be construed as a potential conflict of interest.
